# Multi‐Omics‐Guided Design and Safety Engineering of Nucleic Acid Therapeutics: From Molecular Perturbation to Predictive Toxicology and Precision Translation

**DOI:** 10.1111/cbdd.70378

**Published:** 2026-08-19

**Authors:** Gurjit Kaur Bhatti, Anushka Verma, Komal Devi, Naina Khullar, Inderpal Singh Sidhu, Umesh Chand Singh Yadav, Jasvinder Singh Bhatti

**Affiliations:** ^1^ University Centre for Research and Development Chandigarh University Mohali Punjab India; ^2^ Laboratory of Translational Medicine and Nanotherapeutics, Department of Human Genetics and Molecular Medicine, School of Health Sciences Central University of Punjab Bathinda India; ^3^ Department of Zoology Mata Gujri College Fatehgarh Sahib Punjab India; ^4^ Department of Zoology Sri Guru Gobind Singh College Chandigarh India; ^5^ Special Center for Molecular Medicine Jawaharlal Nehru University New Delhi India

**Keywords:** drug discovery, metabolomics, multi‐omics, NATs, predictive toxicology, proteomics, single‐cell transcriptomics

## Abstract

Nucleic Acid Therapeutics (NATs), including Antisense oligonucleotides (ASOs), small interfering RNAs (siRNAs), and messenger RNAs (mRNAs), are a rapidly developing class of therapeutics capable of specifically regulating previously considered undruggable and inaccessible genes and pathways for modification by small molecules and antibodies. Despite promising results, the utilization of NATs in clinical practice is complicated by the potential off‐target effects, such as activation of the immune response and organ‐specific toxicity, which cannot be effectively predicted based solely on primary structure, chemotype descriptors, or off‐target effects predictors developed in silico. A combination of multiple omics technologies, including proteomics/metabolomics/single‐cell transcriptomics, helps researchers elucidate the interaction of drug compounds with biological targets. This allows for the detection of changes not only at the pathway and cellular level but also early signs of toxicity in parallel. Thus, in this context, this review offers a mechanistic view on the use of multi‐omics strategies for the investigation of NATs‐induced biological effects to analyze the mechanism of action of chemically modified ASOs, siRNAs and mRNA conjugates. The review also discusses case studies in which multi‐omics data have been used to improve therapeutic development. By examining individual layers of molecules separately, a more holistic understanding of treatment mechanisms can be achieved, which is helpful for the discovery of biomarkers and the development of next‐generation nucleic acid drugs.

## Graphical abstract

Multi‐omics (proteomics, metabolomics, and single‐cell/spatial transcriptomics) provides a systems framework to distinguish on‐target efficacy from off‐target and toxicity signatures of chemically modified nucleic acid therapeutics, enabling omics‐guided structure–activity and safety optimisation from discovery to clinical translation.

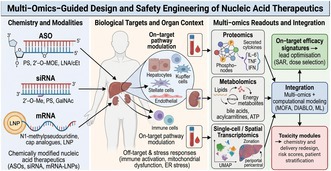

## 1 Introduction

Nucleic Acid Therapeutics (NATs) including ASOs, siRNAs, and mRNA therapies are transforming modern medicine (Belgrad et al. 2024; Kulkarni et al. 2021). These molecules can be easily modified to target pathogenic variants or dysregulated transcripts, making them highly important for precision and personalized medicine (Goetz and Schork 2018). The therapeutic potential of these approaches is highly significant for diseases caused by single‐gene mutations, abnormal splicing, or increased protein production (Di Fusco et al. 2019). To date, over twenty NATs have received worldwide regulatory approval, with many in late‐stage clinical development, representing a rapidly growing therapeutic area (Belgrad et al. 2024). Although NATs have shown remarkable success in early‐phase development, they face substantial challenges in broader clinical adoption and routine therapeutic use (Alhamadani et al. 2022; Meng and Lu 2017; H. Wu et al. 2022). Many of these liabilities arise from hybridization‐dependent off‐target effects on partially complementary RNAs, sequence‐independent interactions with proteins, and innate immune activation (Andersson et al. 2025), immune system activation through pattern recognition receptor signaling (particularly Toll‐like receptors and retinoic acid‐inducible gene‐I signaling RIG‐I) (Hong et al. 2019), hepatotoxicity from lipophilic modifications and conjugates, and systemic toxicities affecting kidney and liver with additional effects reported in other organs (Sun et al. 2016). Chemical modifications such as Phosphorothioate (PS) backbones, Locked Nucleic Acid (LNA), and 2′‐modifications can change how molecules spread in the body, enter cells, and trigger immune reactions however effects are still hard to predict just from their sequences (Aoki et al. 2021; Crooke et al. 2020; Hagedorn et al. 2018; Valentin et al. 2021; Yoo et al. 2004). Even with the rapid development of NATs in clinical practice, the existing validation model remains basic, focusing mainly on transcript‐level measurements such as mRNA knockdown. Even though the measures are important for validating target engagement, they cannot capture the multiple levels of biological effects resulting from the therapeutic approach, including posttranscriptional regulation, rewiring of protein networks, and metabolic adaptation. Studies show that the correlations between transcriptomic changes, protein levels, and their functional properties are poor, especially under stress conditions (Vogel and Marcotte 2012).

Moreover, NATs frequently induce system‐wide perturbations beyond their intended targets, including immune activation, mitochondrial dysfunction, and metabolic reprogramming, which are not adequately reflected in single‐omics analyses (Abdelkader et al. 2023; Crooke et al. 2020). These limitations contribute to poor translational predictability, where candidates demonstrating robust preclinical efficacy fail due to unforeseen toxicities in clinical settings. Therefore, there is a critical need to transition from single‐layer validation approaches toward integrative multi‐omics frameworks that capture the full spectrum of molecular and cellular responses.

The usual drug development approaches have focused on measuring endpoints such as protein expression levels and mRNA knockdown which are insufficient for understanding complex, multi‐system toxicities and variable patient responses that are typical of nucleic acid‐based therapeutics (Abdelkader et al. 2023; Fröhlich 2017). Even though high‐throughput genomic profiling provides useful insights into pathway activity, it is unable to capture posttranscriptional, posttranslational, and metabolic events that influence phenotype and clinical safety (Nguyen et al. 2022; Sanches et al. 2024). As a result, single‐omics studies may miss important factors such as pathway compensation, cell‐type‐specific toxicity, metabolic stress and immune responses, factors that contribute to clinical failure and limit treatment effectiveness across different patient populations (Abdelkader et al. 2023; Nguyen et al. 2022; Sanches et al. 2024; Wu, Chen, and Qin 2025). Multi‐omics approaches combine data from fields such as genomics, transcriptomics, proteomics, metabolomics, and single‐cell analyses to provide a broader picture of how nucleic acid therapies affect biological systems (Y. Wu and Xie 2025). Examining the different layers together helps gain a deeper insight into how these therapies influence various processes at the molecular, cellular, and tissue levels in the body (Sanches et al. 2024). Combining data with advanced computational techniques can effectively identify the toxic effects of treatments and pinpoint cell populations that may cause negative responses. Therefore, this approach supports the development of predictive models that can assess the safety and effectiveness of new therapies (Rouquié et al. 2025; Son et al. 2024). In this review, we discuss how the use of multi‐omics approaches can fast‐track the development of NATs by applying omics tools to study the underlying mechanisms of ASOs, siRNAs, and mRNA conjugates. We have discussed different case studies of how different omics approaches can be used to generate a logical understanding from diverse datasets and showcase how multi‐omics have played a key role in the optimization and development of NATs.

## 2 NATs: Chemistry, Mechanisms, and Known Toxicities

### 2.1 Historical Development and Approved Therapeutics

In the late 1970s, based on Watson‐Crick base pairing principles, the concept of ASOs therapeutics emerged. However, clinical viability improved in 1990s and 2000s when chemical modifications were developed which allowed the cellular uptake, nuclease stability, and enhanced binding affinity (Agrawal 2021; Oberemok et al. 2018). To improve nuclease resistance and enhance plasma protein binding‐mediated tissue distribution, non‐bridging oxygen atoms in the phosphate backbone are replaced with sulfur atoms, generating PS linkages. These linkages, however, promote broad protein binding and can contribute to innate immune activation, including engagement of TLR7, TLR8, and TLR9 in a sequence and chemistry dependent manner. Later, chemical modifications like 2′‐O‐methoxyethyl (MOE), LNAs, and constrained ethyl (cEt) improved therapeutic potency and stability. These modifications reduced innate immune activation with some persistent toxic effects (Pollak et al. 2023; Seth et al. 2008; Swayze et al. 2007). From a medicinal chemistry perspective, NATs occupy a high‐dimensional design space in which backbone, sugar, and base modifications, as well as conjugates and delivery vehicles, jointly determine potency, biodistribution, and toxicity. The systematic variation in PS composition, 2′O modifications, LNA, and cEt moieties generates a set of implicit structure–activity relationships (SAR) that is not easily detected by traditional single‐parameter assays. Multi‐omics assays enable mapping of these SAR; for example, a group of ASOs that differ in their sugar modification patterns can be analyzed by transcriptomics, proteomics, and metabolomics to uncover chemotypes that retain on‐target knockdown without inducing an immune response or liver stress. Similar strategies can be applied to GalNAc valency and linker architectures or to ionizable lipid headgroup chemistries in Lipid Nanoparticle (LNPs). Embedding multi‐omics signatures into SAR analysis transforms nucleic acid optimization from a largely empirical process into a mechanistically informed design‐make‐test cycle.

**FIGURE 1:**
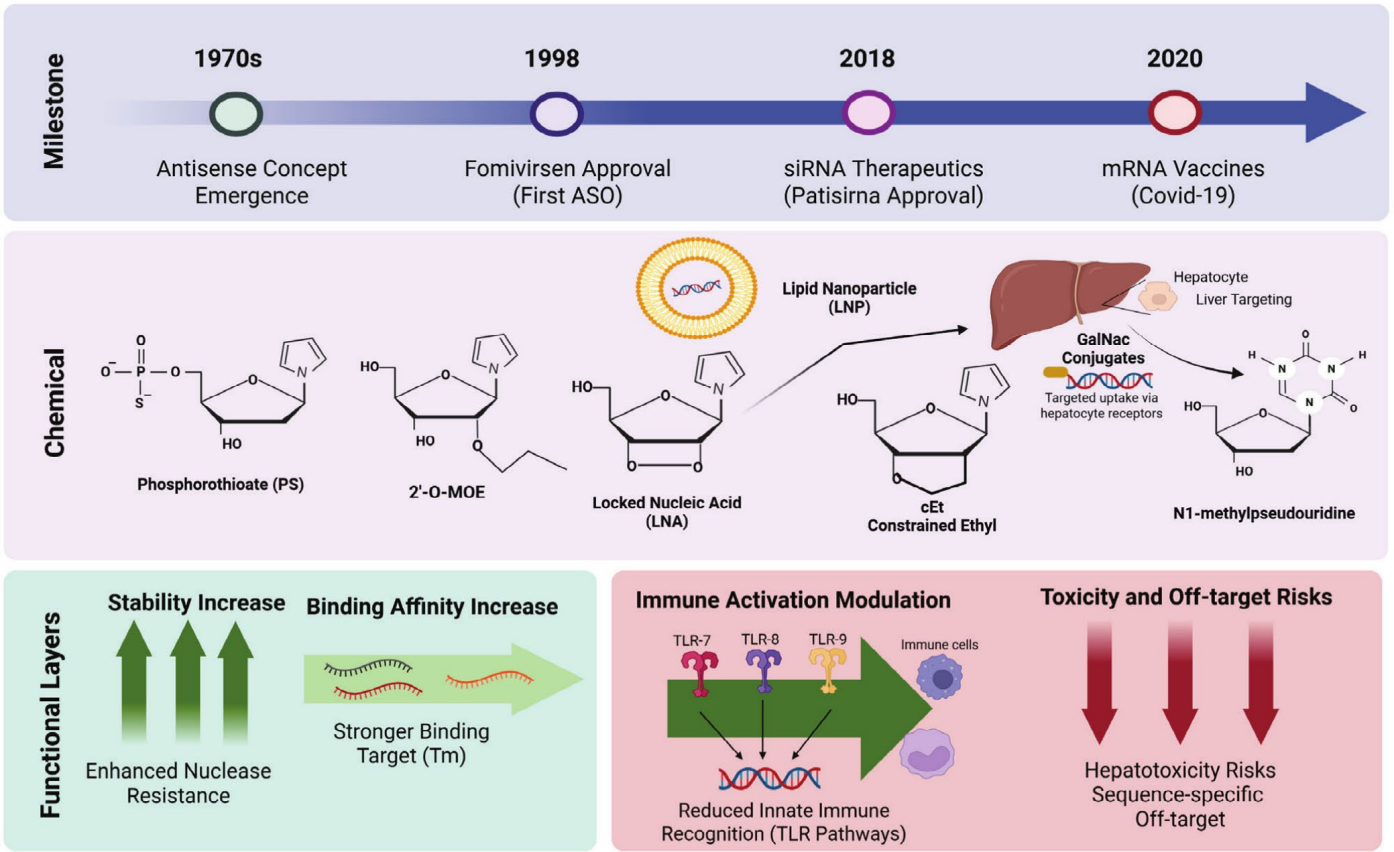
Evolution, chemical optimization, and functional consequences of NATs. This figure incorporates both the evolutionary history of NATs and chemical modifications and their implications in terms of functions. The first panel includes important events in the timeline such as appearance of antisense concepts, approval of Fomivirsen, siRNAs (Patisiran), and mRNA vaccines. In the second panel, different chemical modifications have been shown chronologically, such as PS, 2′‐O‐MOE, LNA, cEt, and N1‐methylpseudouridine, as well as the delivery systems that include LNPs and GalNAc conjugates for hepatocyte targeting. The last panel includes the consequences of the chemical modifications in terms of functionality, which consist of nuclease resistance, increased affinity, Toll‐like receptors (TLR7/8/9) mediated activation, and toxicities, such as hepatotoxicity and off‐target effects.

In the late 1990s, the FDA approved the first antisense therapeutic, Fomivirsen, which targets a specific mRNA type of cytomegalovirus (CMV) IE2 mRNA in AIDS patients (Highleyman 1998). This was a significant breakthrough in clinical research, and since then, more therapies have gained approval, such as Patisiran and Inotersen (Gales 2019; Wood 2018). Both target transthyretin (TTR), which is a protein produced in the liver and binds to thyroxine and retinol (Buxbaum and Reixach 2009). In hereditary ATTR amyloidosis, mutant TTR misfolds and forms amyloid deposits, causing polyneuropathy and cardiomyopathy (Poli et al. 2023; Ruberg et al. 2019). Another therapeutic Nusinersen (Spinraza) targeting the SMN2 gene is developed to treat spinal muscular atrophy (Ottesen 2017). These advancements are an example of the progress made in targeted therapies for serious ailments. mRNA therapeutics were initially explored for cancer vaccines and influenza shots. However, it was during the COVID‐19 pandemic that the field really took off. Researchers then developed vaccines that utilized LNP delivery systems and modified mRNA to enhance stability, minimize immune responses, and boost protein production (Chaudhary et al. 2021). These advances demonstrated the real‐world potential of mRNA‐based therapies, and the key milestones in their development are summarized in Figure 1.

### 2.2 Case Study 1: Patisiran (ONPATTRO) Proteomics‐Driven Biomarker Discovery and Target Validation

Patisiran, an LNP‐formulated siRNA that targets TTR mRNA, was approved by the FDA in 2018 for the treatment of hereditary ATTR amyloidosis with polyneuropathy (hATTR‐PN). Although decreased TTR levels in the serum confirmed target engagement, the most compelling multi‐omics impact came from the proteomics sub study conducted during the phase 3 APOLLO trial (NCT01960348). In this study, serum levels of over 1000 proteins were measured in 136 patisiran‐treated hATTR‐PN patients at baseline, 9 months, and 18 months, alongside placebo‐treated patients and healthy controls. Among the 66 proteins that showed significant differences between the patisiran and placebo groups, neurofilament light chain (NfL), a structural neuronal protein and a sensitive biomarker of axonal injury, showed the most significant treatment associated change. Critically, NfL levels increased in the placebo arm (reflecting progressive neurodegeneration) while decreasing significantly in the patisiran group, with the overall proteome profile of treated patients tending toward that of healthy controls at 18 months (Adams et al. 2025; Moriyama et al. 2026; Yang 2019). Proteomics based findings had important translational implications, as NfL subsequently emerged as a promising biomarker for monitoring neuroaxonal injury and treatment response in ATTRv amyloidosis. Besides measuring TTR knockdown alone, NfL provided an additional means to evaluate the biological impact and therapeutic efficacy of patisiran. Moreover, additional proteomic analyses of the blood of ATTR patients revealed several molecular pathways affected by the disease, including inflammation, complement pathway activation, oxidation of TTR and ApoE proteins, and protease activity—none of which can easily be detected by transcriptomics. Overall, the results provided evidence that the effects of patisiran extended beyond target engagement, through modulation of other proteins, suggesting disease modification. This suggests that the therapeutic effect of patisiran extends beyond targeting the causative gene, as indicated by proteomic analysis (Ioannou et al. 2023). Key lessons from this multi‐omics guided drug development were (i) Plasma proteomics can reveal pharmacodynamic biomarkers complementary to target mRNA knockdown, providing stronger proof of therapeutic effects; (ii) Longitudinal multi‐protein analysis can reveal whether the therapeutic has shifted the disease associated proteome back towards a healthy molecular state; and (iii) Proteomics‐based biomarkers like NfL may help patient stratification and monitoring of disease and treatment response in clinical trials (Moriyama et al. 2026).

### 2.3 Case Study 2: LNA‐Modified ASOs‐Multi‐Omics Revealing Hepatotoxic Chemotypes and Guiding Chemical Redesign

Early generation LNA gapmers were associated with unexpected hepatotoxicity in rodents, and conventional cytotoxicity assays did not reliably predict this liability (Burdick et al. 2014). In a landmark sequence toxicity study, toxicity was observed with some LNA gapmers targeting genes such as Apoc3, Crtc2, and GR. Most importantly, the relationship observed was between the motifs, namely TGC and TCC, rather than the gene in question. The toxic motifs were found to have a higher affinity for liver cell proteins and for signaling pathways that included P53 and Nrf2. Subsequent studies showed that some LNA gapmer hepatotoxicity can also involve RNase H1‐dependent off‐target cleavage of very long pre‐mRNAs. From a chemical perspective, toxicity may be reduced by varying the gapmer structure or replacing some LNA nucleotides with alternative chemistries, such as 2′‐OMe, MOE, or cEt. Nevertheless, depending on the sequence, there could be consequences for both potency and toxicity (Swayze et al. 2007). Later, Inotersen reached the clinical stage with additional attention paid to thrombocytopenia and kidney toxicities (Brannagan et al. 2022).

Despite impressive clinical successes, NATs exhibit certain side effects and toxicity patterns related to chemistry, modification patterns, delivery vehicles, and conjugates (Debacker et al. 2020; Hagedorn et al. 2013). PS‐modified ASOs have been shown to increases risk of liver damage. Increase in liver enzyme transaminases, fat buildup in liver and hepatocyte necrosis are commonly seen. These issues are more common in DNA sequences with GGG repeats and when high levels of ASO are present in liver (Hagedorn et al. 2013; W. Shen et al. 2018). The therapeutic potency is increased by strengthening the binding to the target RNA by locked nucleic acids. However, the risk of hepatotoxicity remains high due to abnormal protein binding, mitochondrial dysfunction, and activation of innate immune responses (Burdick et al. 2014; Burel et al. 2016; W. Shen et al. 2018; Swayze et al. 2007).

To deliver the target more efficiently to liver cells, GalNAc conjugates are designed, which allow it to be recognized by the asialoglycoprotein receptor. This approach typically makes treatments more tolerable since it limits exposure to the rest of the body. There are still risks, such as off‐targets and dose‐related liver damage (Debacker et al. 2020; Janas et al. 2018). Off‐target effects, such as random base pairing with complementary RNA molecules and protein interactions, are a major problem in therapeutic research. Also, traditional in vitro cytotoxicity assays are unreliable for predicting in vivo hepatotoxicity. This limitation can be addressed by using a broader approach, like multi‐omics (Cheng et al. 2011; Kamola et al. 2017; Ruan et al. 2025; Scharner et al. 2020).

When unmodified RNA or dsRNAs are present as contaminants in mRNA therapies with LNP, it can activate innate immune response through endosomal TLR7/8 and cytosolic RNA sensors such as RIG‐I and MDA5. It can trigger a systemic interferon response and increase the cytokine levels, particularly at high doses or in immunocompromised individuals. The composition of the LNP is one of the determinants of the distribution of therapies throughout the body. Factors such as ionizable lipids, PEGylation density, and surface modification will determine whether particles aggregate in the liver or spleen and whether the cells that take them up are lymphoid or parenchymal (Lee et al. 2023; Wu, Zhang, and Ni 2025; Zhivaki et al. 2023). Different classes of NATs and their key features are summarized in Table 1.

**TABLE 1:** Classes of NATs and their key features.

Therapeutic type	Mechanism of action	Common chemical modifications	Delivery systems	Approved examples	References
ASOs	Bind complementary mRNA leading to RNase H‐mediated degradation or splice modulation	PS backbone, 2′‐O‐MOE, LNA, cEt	GalNAc conjugates, LNP	Fomivirse, Inotersen, Nusinersen	Crooke et al. (2021); Dhuri et al. (2020)
siRNA	RNA interference via RNA‐Induced Silencing Complex (RISC) leading to target mRNA cleavage	2′‐O‐methyl, PS, LNA	LNP, GalNAc conjugates	Patisiran	Hu et al. (2020); Shevelev et al. (2025)
mRNA Therapeutics	Translation of encoded protein after delivery to cells	N1‐methylpseudouridine, cap analogs	LNPs	mRNA vaccines	Kim et al. (2022); Nance and Meier (2021)

## 3 Conceptual Framework: Multi‐Omics as Integrated Perturbation Analysis

**FIGURE 2:**
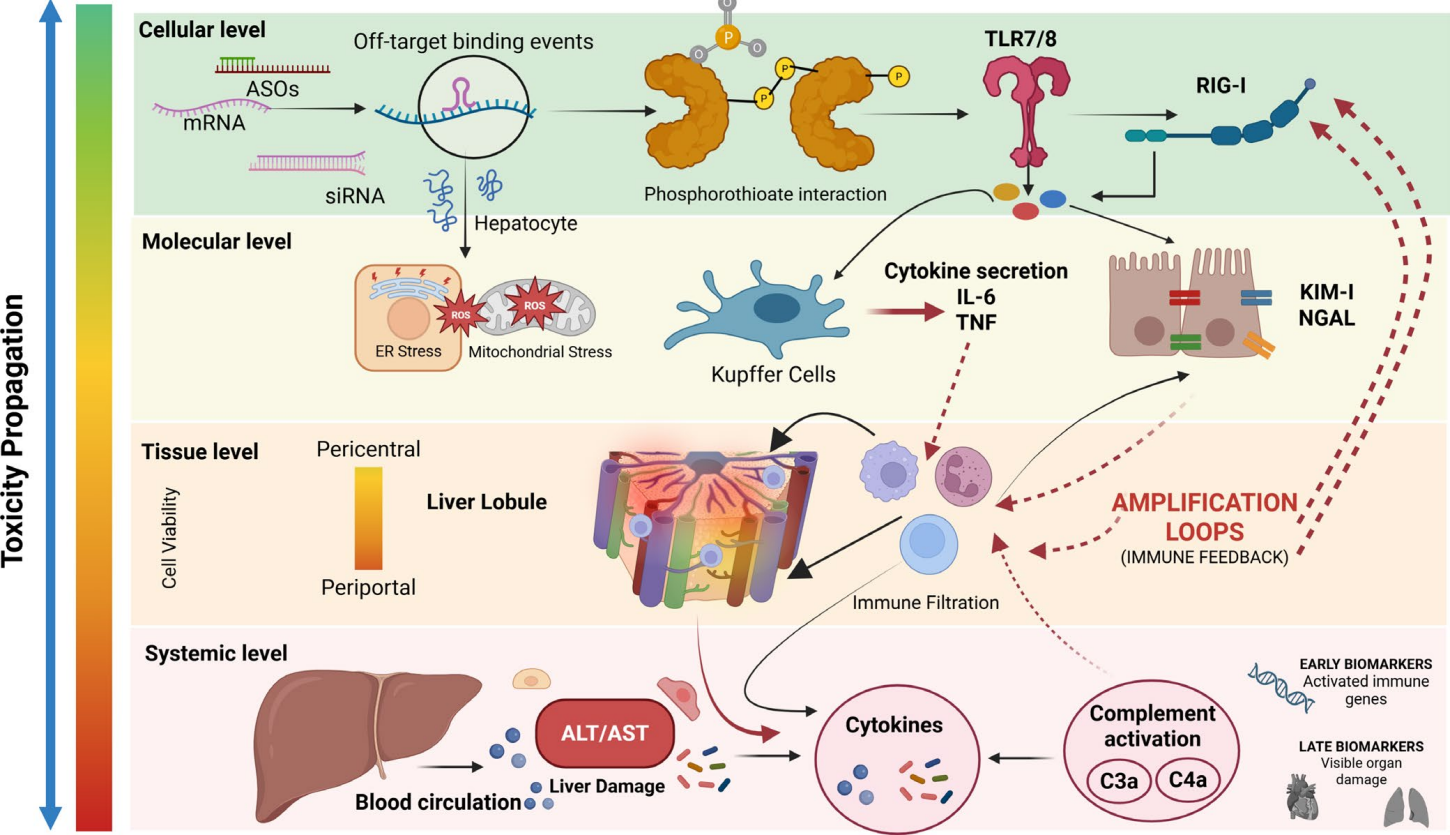
Cross‐scale propagation of toxicity from molecular events to systemic outcomes. This shows the progression of toxicity propagated by NATs from one biological scale to another. In terms of molecular events, off‐target RNA binding and protein interactions caused by PS lead to activation of innate immune receptors like TLR7/8 and RIG‐I. This results in the occurrence of certain events within the cell such as ER stress, mitochondrial ROS production and cytokine secretion by Kupffer cells, as well as damage to the renal tubular cells (KIM‐1, NGAL). On the tissue scale, these result in zonated injury of the liver and immune cell infiltration due to amplification of the process by inflammatory feedback loops. Systemically, they manifest in terms of circulating markers like ALT/AST, cytokines, and complement activation (C3a, C4a).

Nucleic acid therapies create changes in RNA, protein, and metabolites at the cellular, tissue, and organ levels. With multi‐omics approaches, we can observe a series of secondary effects that spread through various signaling networks, metabolic pathways, and immune responses simultaneously (Chen, Wang, et al. 2023). When analyzed together, transcriptome, proteome, and metabolome data help researchers better understand how therapies affect different organs and immune cells. This combined approach helps identify true on‐target therapeutic effects from unwanted off‐target effects or toxicity. So, a broader view of drug development helps in developing a deeper mechanistic understanding, leading to improved treatment and more effective safety management (Andersson et al. 2025; Yoshida et al. 2019). Multi‐omics validation distinguishes three mechanistically and functionally separable phenotypes: on‐target effects, off‐target effects, and toxicity signatures. The hierarchical propagation of these molecular perturbations into cellular, tissue, and systemic toxicity phenotypes is illustrated in Figure 2.

### 3.1 On‐Target Signal

Expected changes in mRNA or protein levels and their downstream pathway components are consistently seen across transcriptomic, proteomic, and metabolomic levels within cellular populations. These observations help in confirming that the therapy is working at its designated molecular target and support the dose response modeling and efficacy prediction (Watts and Corey 2012).

### 3.2 Off‐Target Effects

Unexpected changes in nontarget RNAs or proteins arising from sequence similarity, structure‐dependent protein interactions, or incorrect mRNA splicing lead to uncertain omics signatures in unintended cellular populations. Detection of these off‐target effects early in the preclinical phases helps researchers improve treatments by changing the delivery approaches, optimizing sequences, and redesigning through chemical modification (Ruan et al. 2025; Yoshida et al. 2019).

### 3.3 Toxicity Signatures

During stress, cells activate various pathways, including innate immune activation, mitochondrial dysfunction, ER stress, and apoptosis. These conditions are usually more significant in untargeted or low‐expressing cell populations such as Kupffer cells, macrophages, and renal tubular cells. Through multi‐omics, these toxicity signatures can be detected, enabling mechanism‐based interventions and patient stratification strategies (Andersson et al. 2025; Cai et al. 2022).

Importantly, multi‐omics phenotypes should be interpreted in relation to the specific chemical series under investigation, as their biological significance depends on the chemical context rather than representing universal molecular perturbations. Each backbone modification pattern, sugar chemistry, GalNAc topology, or ionizable lipid scaffold can be thought of as a distinct ‘chemical node’ that imprints a characteristic omics fingerprint on target tissues and circulating biomarkers. The integration of molecular fingerprints, pathways, and cell‐type networks allows chemists and biologists to make informed design decisions. These include choosing suitable backbone chemistries, minimizing Kupffer cell activation, and designing LNPs that reduce zonation‐dependent hepatotoxicity while preserving efficient delivery. Chemistry‐based interpretation of multi‐omics datasets is required to derive meaningful design principles for next‐generation nucleic acid drugs.

## 4 Proteomics and Metabolomics for On‐Target Validation, SAR, and ADMET Optimization

Multi‐omics provides a robust framework for extending the classical ADMET (absorption, distribution, metabolism, excretion, and toxicity) paradigm to NATs. Unlike small molecules, NATs utilize mainly carrier‐mediated uptake, endosomal trafficking, and intracellular processing, resulting in highly complicated distribution and transformation. The use of proteomics and metabolomics enables the definition of ADME fingerprints that are unique to each chemotype, reflecting patterns of specific endocytic receptor engagement, lysosomal stress, or alterations in bile acid and lipid transformation. When combined with plasma and tissue biomarker readouts, the data sets allow calculating toxicity scores that, in addition to classical parameters like maximum tolerated dose or ALT/AST threshold, become an important quantitative criterion. In this way, multi‐omics becomes a perfect ADMET platform that enables deprioritizing chemotypes with the signs of early mitochondrial dysfunction, complement activation, or renal tubular injury without GLP toxicology studies.

### 4.1 Role of Proteomics in Nucleic Acid Therapeutic Validation

The direct quantitation of mRNA knockdown or increased mRNA translation of protein can be confirmed with proteomics using mass spectrometry. It is very important to connect transcriptional regulation with its phenotypic consequences using this method. Protein quantification at different doses will provide information on the drug's effect. The dose response curve, ED50, and other drug effects can be obtained through this quantification. Now, several hundred proteins can be measured simultaneously using new proteomic techniques, including data‐independent acquisition (DIA) and tandem mass spectrometry. It allows analysis of multiple pathways that are not directly related to the main target (T. T. Lin et al. 2023). Investigating the effect arising from target suppression may reveal how the disruption spreads through the connected signaling network. This way, it is possible to identify the pathway components, such as kinases, transcription factors, and regulatory proteins, whose behavior indicates pathway dysfunction. For example, when ASO treatment reduces PCSK9 levels in liver cells, an increase in low‐density lipoprotein receptor (LDLR) levels is expected. If there are any deviations from these anticipated changes, it may suggest accidental side effects (Bao et al. 2024; Lagace 2014). Phosphoproteomics and ubiquitinomics have shown that when a specific pathway is inhibited, a compensatory pathway gets activated. This can reduce the effectiveness of treatments and contribute to resistance to therapies. For example, when a particular kinase is knocked down, it leads to the activation of other related kinases or the activation of alternative survival mechanisms through phosphorylation. Early recognition of these responses' aids in the design of more effective combination therapies that can target multiple pathways simultaneously and improve treatment outcomes (Huang and White 2008).

Protein phosphorylation at thousands of sites is measured at once using phosphoproteomics. This application offers valuable insights into the activation states of signaling pathways before changes in protein levels. This is important for understanding functional shifts in signaling (Huang and White 2008). In the context of nucleic acid therapies, phosphoproteomics can serve the following purposes:

1. It can detect the activation or inhibition of kinases within a short time after treatment. Providing early indicators of biological activity that may occur before changes in mRNA and protein levels (Higgins et al. 2023).
2. It helps map the relationship between dose and response through Phosphoproteomics profiling, allowing the identification of specific kinase targets engaged at therapeutic doses (Schmidlin et al. 2019).
3. It can identify stress signaling pathways, such as those related to DNA damage (kinase such as ATM, ATR, and CHK2), the unfolded protein response (involving eIF2α and IRE1), and AMPK‐mediated responses to energy stress, all of which can indicate potential toxicity (Faca et al. 2020; Schlam‐Babayov et al. 2021).

Ubiquitinomics and acetylomics extend this perspective by profiling lysine ubiquitination and acetylation. These posttranslational modifications affect protein stability, localization, and function and provide additional layers of pathway regulation (Kozuka‐Hata et al. 2020; Steger et al. 2022).

Off‐target ASOs may suppress or induce unwanted proteins, but with proteome‐wide approaches such modifications can be detected and termed as outliers from expected pathway changes (X. Liu et al. 2021). mRNA splicing variation can produce protein isoforms due to incorrect start codons or out‐of‐frame exons, leading to the production of truncated or misfolded proteins. These can be detected using proteomic approaches (X. Liu et al. 2021). HLA class I and II immunogenicity can be evaluated through peptide profiling, which can be performed using immunoprecipitation and mass spectrometry. This approach helps identify novel peptides from edited proteins or misfolded products that may activate T‐cell responses (Creech et al. 2018).

Cytokines, chemokines, acute‐phase reactants, and tissue damage markers, are examples of secreted proteins which reflect systemic pathway activation and organ toxicity compiled in Table 2. This plasma proteomics can be used to identify cytokine detection, such as Interferon alpha (IFN‐α), Interferon beta (IFN‐β), Tumor Necrosis Factor alpha (TNF‐α), Interleukin 6 (IL‐6), and Interleukin 12 (IL‐12), shows activation of innate immune pathways. These responses are usually controlled by signaling through pattern‐recognition receptors, such as Toll‐like receptors (TLRs), RIG‐I, and the cGAS‐STING pathway (Kawai and Akira 2008; Zergoun et al. 2022). Tissue‐specific proteins including liver transaminases (ALT, AST) (Åberg et al. 2025), kidney injury molecules (KIM‐1, NGAL) (Zdziechowska et al. 2020), cardiac troponins (Gaze and Collinson 2005), and neuron‐specific enolase are indicators of organ‐specific toxicity. Sometimes ASO chemistries can directly activate the complement pathway, as shown by plasma C3a, C4a, and other complement fragments depending on sequence and formulations (Shen et al. 2016).

**TABLE 2:** Major toxicity mechanisms of NATs.

Toxicity type	Molecular cause	Biological consequences	Detection methods	References
Off‐target RNA binding	Partial sequence complementarity	Unintended gene silencing	Transcriptomics, proteomics	Ruan et al. (2025); Watts and Corey (2012)
Innate immune activation	TLR7, TLR8, TLR9 or cGAS‐STING signaling	Interferon production, cytokine release	Cytokine profiling, scRNA‐seq	Guo, Lu, et al. (2025); Mosallanejad and Kagan (2022)
Hepatotoxicity	Accumulation of modified oligonucleotides in hepatocytes	Elevated ALT/AST, lipid accumulation	Proteomics, metabolomics	Hagedorn et al. (2013); Moreno‐Torres et al. (2022)
Complement activation	Interaction with plasma proteins	Hypersensitivity reactions	Plasma proteomics	Henry et al. (1997)
Mitochondrial dysfunction	Oxidative stress and metabolic disruption	Energy imbalance, apoptosis	Metabolomics	Bayir and Kagan (2008); Korotkov (2023)

### 4.2 Proteomics Technologies and Methodologies

Modern proteomics integrates multiple complementary acquisition strategies for comprehensive and unbiased analysis of the proteome. In data‐dependent acquisition (DDA), sampling is focused on the most abundant proteins. Enabling deep proteome coverage, but it is potentially biased toward highly abundant species. This approach offers strong posttranslational modifications (PTMs) detection due to its greater fragmentation depth (T. T. Lin et al. 2023). In DIA, peptides are systematically fragmented in a predefined mass‐to‐charge window. This provides global quantification and improved reproducibility across samples. DIA allows detection of low‐abundance proteins missed by DDA (Y. Du et al. 2021).

Parallel Reaction Monitoring (PRM) is a sensitive method that is used to measure specific peptides in complex mixtures. It is useful in detecting biomarkers as it can quantify peptides. A major advantage is that it captures complete result of mass spectrometry analysis and reduces interference. This makes it ideal for identifying biomarkers from discovery proteomics in plasma, urine, or tissue. It is useful in validating low abundance peptides for monitoring, such as in cancer or metabolic disorders while maintaining reliable results across different samples (Peterson et al. 2012).

Proteome coverage and depth improve through targeted enrichment. Techniques such as titanium dioxide (TiO_2_) affinity chromatography, immobilized metal affinity chromatography (IMAC), and phospho‐specific antibodies are key tools for isolating phosphorylated peptides. These techniques allow for large‐scale detection and quantification of phosphorylation events (Huang and White 2008). Anti‐ubiquitin antibody enrichment, combined with diGly peptide capture, offers detailed view into protein ubiquitination. These methods allow mapping of modification sites with high precision (Zafar et al. 2024). Depletion of abundant proteins, such as albumin, transferrin, and immunoglobulins, reveals lower‐abundance components. Such enrichment improves sensitivity and supports the identification of disease‐related biomarkers (Tu et al. 2010).

### 4.3 Role of Metabolomics in Efficacy and Safety Prediction

Metabolomics provides sensitive downstream phenotypic readouts of pathway regulation, integrated with the effects of multiple genes and proteins. This allows the systems‐level evaluation of functional outcomes (Pan et al. 2024). For NATs metabolomics can reveal the following: Metabolomics helps understand changes in the body's metabolism induced by the suppression of specific targets. For example, ASO‐mediated suppression of PCSK9 mRNA increases LDL receptor levels, enhances hepatic cholesterol uptake, decreases plasma LDL‐cholesterol, and alters liver cholesterol metabolism, patterns detectable through lipidomics (Gupta et al. 2010; Visser et al. 2012). Using stable isotope‐labeled substrates and metabolomics enables us to track how metabolites affect specific pathways in the body. This helps us understand whether reducing the levels of a particular protein decreases the flow of metabolites through that pathway or whether the body can adjust to support steady flow even when we have suppressed that protein. For instance, inhibition of a glycolytic enzyme, decreased glucose consumption, and decreased glycolytic activity are expected and could be measured by checking whether labeled glucose‐based carbs appear in the metabolic products being tracked (Creek et al. 2012; Wang et al. 2022). Metabolomics can uncover unexpected links in cellular metabolism, showing how changes at the gene level influence cell behavior. For example, when the mTOR pathway is inhibited, some cancer cells begin to depend heavily on glutamine for survival. By identifying these metabolic weaknesses through metabolomics, targeted therapies can be developed to improve treatment outcomes (Adebayo Michael et al. 2019; Tanaka et al. 2015).

Chemically modified ASOs and siRNAs exhibit characteristic metabolic perturbations linked to their chemistries. ASOs with PS backbones accumulate in hepatocytes and interfere in bile acid metabolism, lipid homeostasis, and cellular iron handling. These changes are detectable through lipidomics and metabolic profiling (Gilar et al. 1997; Miller et al. 2018). Some ASO chemistries can affect nucleotide metabolism during their intracellular processing and degradation. This generates intermediates that interact with endogenous purine and pyrimidine pools. These changes are detected using mass spectrometry‐based metabolomics, helping identify therapy‐induced metabolic imbalances (Kilanowska and Studzińska 2020; Shadid et al. 2021).

LNP delivery vehicles accumulate in hepatocytes because ApoE‐coated particles interact with LDLR and VLDLR receptors in the liver. This interaction can change hepatic lipid metabolism, glycolysis, and mitochondrial function. These changes can be detected through lipidomics, which measures triglycerides, phospholipids, and cholesterol esters. Energy metabolism assays, such as extracellular flux analysis and acylcarnitine and ATP measurements, help monitor glycolysis and oxidative phosphorylation. Recent studies suggest that these metabolic readouts should be included in preclinical testing of LNPs, as factors like lipid composition, particle charge, and protein corona formation can influence liver uptake and metabolic responses (Hosseini‐Kharat et al. 2025; Paroor et al. 2025; Su et al. 2024). Many NATs affect the cellular redox balance by inducing TLR‐mediated ROS production or mitochondrial dysfunction. These changes can be quantified using glutathione redox ratios, lipid peroxidation products, and additional oxidative stress biomarkers (T. H. Chen et al. 2024; Ren et al. 2021).

Specific metabolite patterns can serve as early biomarkers of efficacy and toxicity. Metabolite changes reflecting target pathway inhibition often appear soon after treatment and can predict therapeutic efficacy more effectively than gene expression changes alone. For example, reduced hepatic CoA levels and changed fatty acid oxidation patterns indicate effective suppression of SREBP‐mediated lipogenesis (Zhao et al. 2022). Metabolite signatures of mitochondrial dysfunction and other indicators like oxidative stress, ER stress, and immune activation can appear weeks before clinical signs of toxicity. This allows a period for early intervention. These include glutathione depletion, increased acyl‐carnitines, elevated tricarboxylic acid cycle intermediates, and modified amino acid ratios (McGill et al. 2014; Sillé and Hartung 2024).

### 4.4 Metabolomics Technologies and Data Integration

Metabolomics employs complementary analytical methods. Liquid Chromatography‐Mass Spectrometry (LC–MS) technique quantifies a wide range of polar and semipolar metabolites. It is commonly used to measure amino acids, organic acids, and nucleotides with high sensitivity and a wide dynamic range (Astarita et al. 2023). Lipidomics focused on lipid analysis; lipidomics identifies classes such as phospholipids, triglycerides, cholesterol esters, and sphingolipids. These lipids can be identified using LC–MS or gas chromatography–mass spectrometry (GC–MS) (Rakusanova and Cajka 2024). Nuclear Magnetic Resonance (NMR) Spectroscopy offers unbiased metabolite profiling and simultaneous quantification. It is highly effective for analyzing plasma and tissue samples (Jang et al. 2018). Untargeted Metabolomics approach measures thousands of metabolites across pathways. It helps identify unexpected metabolic changes and discover potential biomarkers (Sillé and Hartung 2024). Targeted Metabolomics focuses on specific, predefined metabolites. It is mainly used for biomarker validation, pathway analysis, and clinical studies (Jang et al. 2018).

## 5 Single‐Cell and Spatial Omics for Cell‐Type–Resolved Safety Engineering

### 5.1 Determining Cell Type‐Specific On‐Target Engagement

**FIGURE 3:**
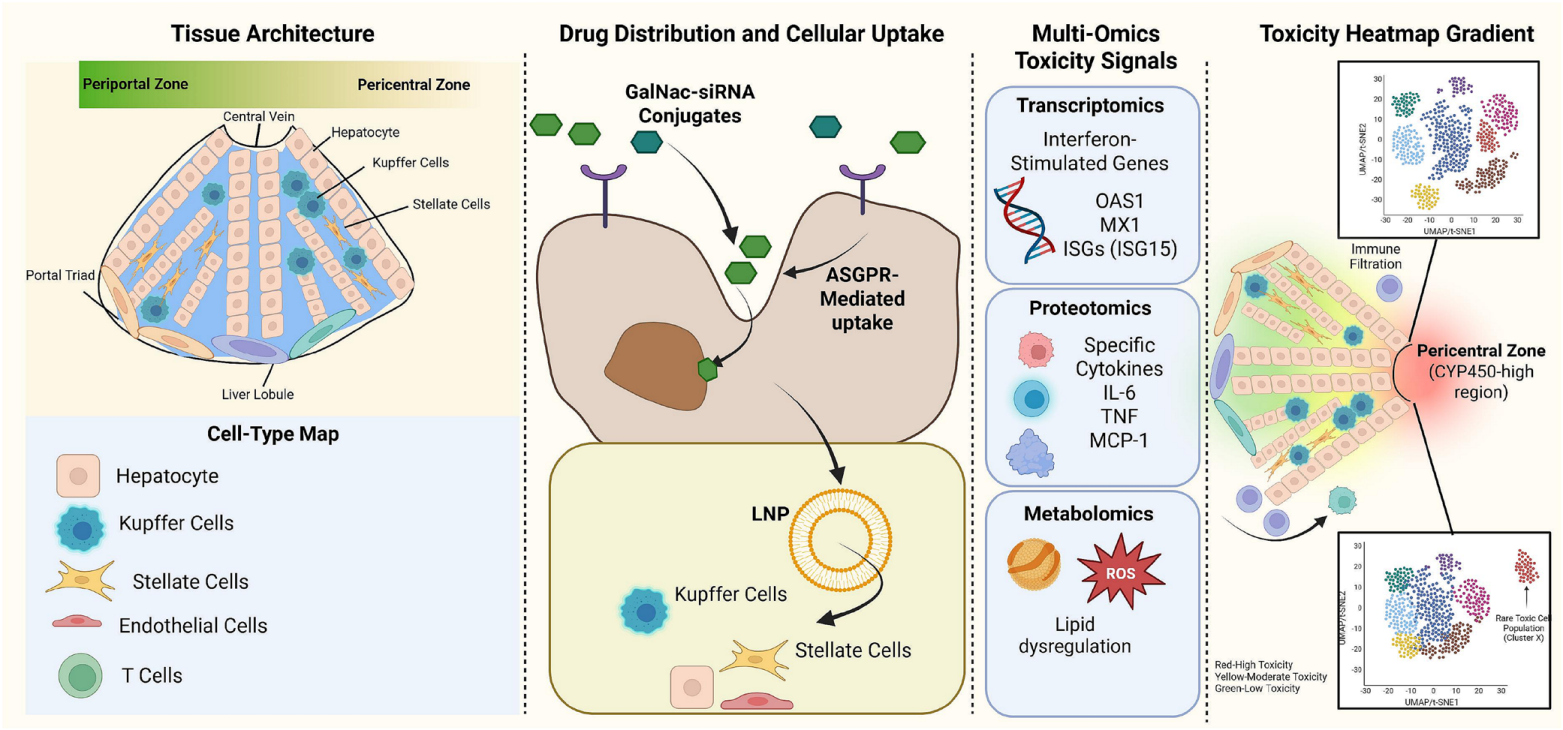
Spatio‐Cellular Toxicity Atlas of NATs. This multi‐panel figure depicts spatial, cellular, and molecular heterogeneity in responses to NATs. (A) Lobule architecture of the liver indicating zonation around the periportal and pericentral zones and their cell types. (B) Map of cell‐types indicating hepatocytes, kupffer cells, stellate cells, endothelial cells, and immune cells. (C) Distribution of drugs wherein hepatocytes selectively take up GalNAc‐siRNA through ASGPR, while lipid nanoparticles are widely distributed in other cell types. (D) Multi‐omics toxicity signals, including transcriptomic interferon stimulated genes, proteomic cytokines (IL‐6, TNF, MCP‐1), and metabolomic indicators such as ROS and lipid dysregulation. (E) Spatial toxicity gradients demonstrating pericentral vulnerability and immune infiltration, complemented by single‐cell RNA‐seq clustering to identify rare toxic cell populations.

Single‐cell RNA sequencing (scRNA‐seq) and emerging single‐cell multi‐omics (RNA, protein, and chromatin) reveal where and in which cell states therapeutics achieve target engagement across heterogeneous cell populations (Van de Sande et al. 2023). Liver cells, including hepatocytes, Kupffer cells, hepatic stellate cells, and endothelial cells, exhibit marked differences in ASO and siRNA uptake, as summarized in Figure 3. Therefore, it helps identify which cell populations are exposed to the therapeutics. GalNAc‐conjugated siRNAs usually show selective uptake by hepatocytes, and uptake by immune cells may indicate failure of the delivery system (Debacker et al. 2020; Payen et al. 2021). Cellular populations with high mRNA expression levels show significant target knockdown and similar changes in downstream gene expression. Such cells with low target expression remain unaffected. Deviations from this pattern may indicate off‐target effects or toxicities that depend on the cell type (Van de Sande et al. 2023). scRNA‐seq helps in identifying unexpected subcellular populations in hepatocytes. Some subtypes show pronounced metabolic stress even though effective target knockdown, which indicates toxicity mechanisms independent of target engagement (Coassolo et al. 2023; Sanchez‐Quant et al. 2023). scRNA‐seq provides sensitive detection of toxicity phenotypes in rare cell populations:

Early activation of TLR signaling is indicated by transcriptional signatures that include interferon‐stimulated genes (IFIT1, IFIT2, IFIT3, IFIT5, OAS1‐3, OASL, MX1, MX2, PKR), chemokines (CCL2, CCL3, CCL4, CXCL10, CXCL11), and pro‐inflammatory cytokines (IL‐6, TNF). These together serve as indicators of TLR‐dependent activation in innate immune cells, such as macrophages, dendritic cells, and neutrophils (Goodchild et al. 2009; Stubbington et al. 2017). Some toxicities occur in small, rare cellular subpopulations, such as proximal tubular epithelial cells, which display transcriptomic injury signatures (Xu et al. 2025). Microglial cells undergo activation and pro‐inflammatory polarization in CNS‐targeted therapies, changes that are detectable only by scRNA‐seq (Esaulova et al. 2020). Damaged cells show transcriptomic signatures of apoptosis, for example activation of caspase‐related genes, death receptor signaling pathways, and dysregulation of cell cycle checkpoints. Detection of these signatures in nontargeted cell populations suggests off‐target toxicity (Van de Sande et al. 2023).

### 5.2 Spatial Transcriptomics for Anatomical Toxicity Localization

Combining bulk transcriptomics or scRNA‐seq with spatially resolved profiling reveals compound accumulation and toxicity patterns in periportal versus pericentral liver regions, reflecting zonated detoxification capacity and metabolic differences. For example, therapeutics display various patterns of toxicity in specific zones with CYP450 expression patterns and metabolic enzyme distribution (Umbaugh et al. 2021). How injured cells communicate through paracrine signaling and initiate downstream cascades can be studied by spatial transcriptomics. For example, hepatocyte injury can activate Kupffer cells via damage‐associated molecular patterns and further amplify inflammatory responses (Ahmed et al. 2021; Slevin et al. 2020).

### 5.3 Single‐Cell Transcriptomics Technologies and Platforms

#### 5.3.1 High‐Throughput scRNA‐Seq Methods

10× Genomics is a droplet‐based microfluidic platform that allows high‐throughput encapsulation and barcoding of thousands of individual cells per sample (Table 3). This method allows large‐scale transcriptomic profiling with high reproducibility and standardized workflows (Van de Sande et al. 2023). Smart‐seq2 is a full‐length transcript sequencing method that provides enhanced sensitivity (Table 3). For detecting splice variants, transcript isoforms, and low‐abundance transcripts compared with 3′ or 5′ end counting approaches (Van de Sande et al. 2023).

**TABLE 3:** Contributions of different omics technologies to nucleic acid therapeutic evaluation.

Omics layer	Biological information	Key technologies	Applications in therapeutics	References
Transcriptomics	Gene expression changes	RNA‐seq, scRNA‐seq	Target knockdown detection	Saju et al. (2025); Van de Sande et al. (2023)
Proteomics	Protein abundance and signaling pathways	LC–MS/MS, phosphoproteomics	Pathway validation, immune activation	Wu et al. (2023)
Metabolomics	Cellular metabolic state	LC–MS, GC–MS, NMR	Functional pathway assessment	Zeki Ö et al. (2020)
Single‐cell omics	Cell‐type specific responses	10× Genomics, Smart‐seq2	Toxicity detection in rare cells	Van de Sande et al. (2023)
Spatial transcriptomics	Tissue‐level organization	Visium, MERFISH	Localization of toxicity	Zhang et al. (2022)

#### 5.3.2 Single‐Cell Multi‐Omics

CITE‐seq is a multimodal technique that includes scRNA‐seq with oligonucleotide‐tagged antibodies. This technique allows quantification of gene expression and surface protein abundance in individual cells at the same time (Van de Sande et al. 2023). scATAC‐seq assay profiles chromatin accessibility across the genome. This technique provides insights into regulatory elements, transcription factor activity, and dynamic changes in cell states (Gur and Hughes 2025). Single‐cell metabolomics combines single‐cell isolation strategies with mass spectrometry‐based metabolomic analysis. It enables cell‐type‐specific metabolic profiling and investigation of metabolic heterogeneity (Sanchez‐Quant et al. 2023).

## 6 Computational Integration and Machine Learning for Candidate Prioritization

### 6.1 Unsupervised Integration Methods

**FIGURE 4:**
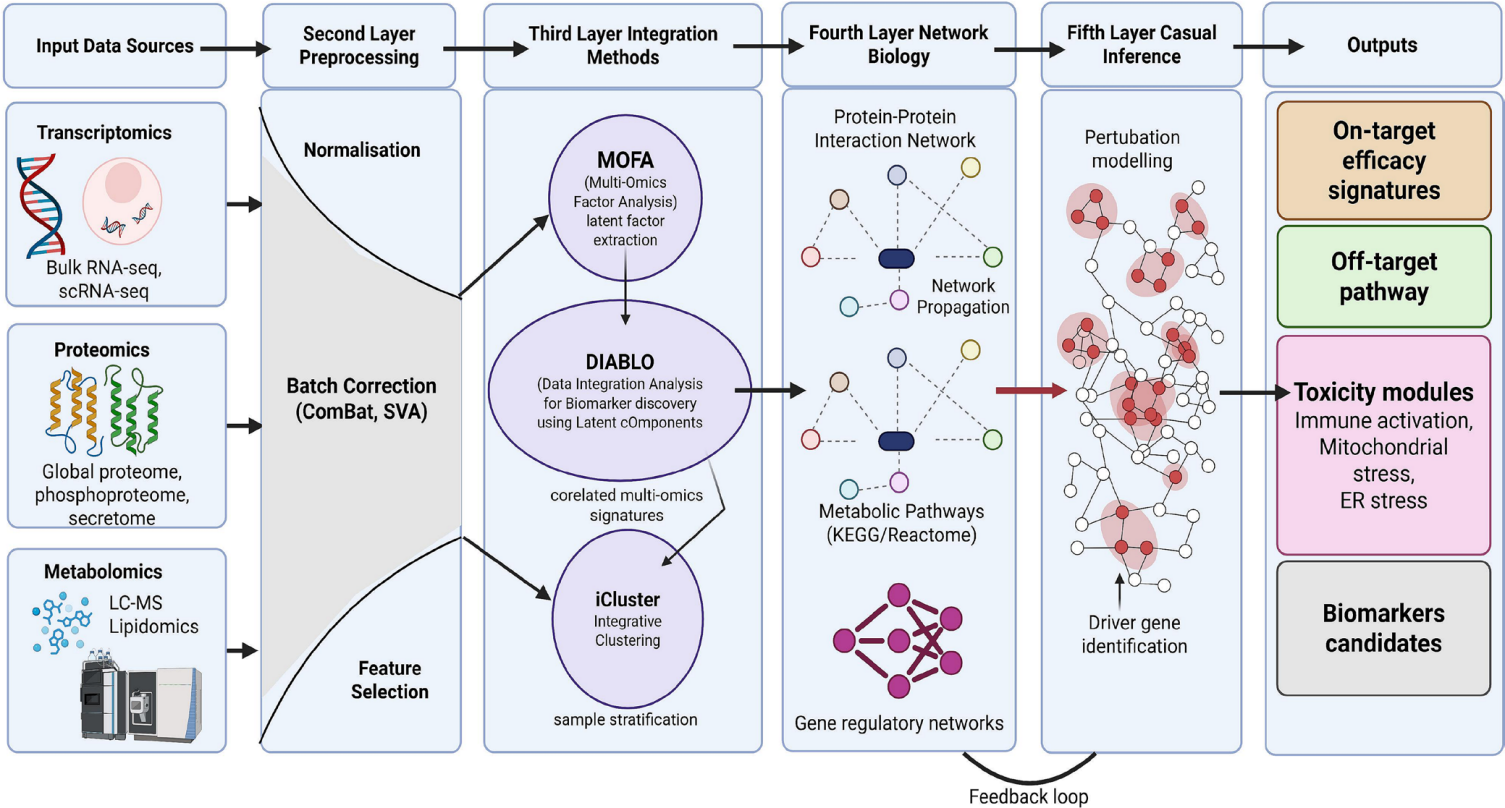
Integrated multi‐omics pipeline for mechanistic inference and biomarker discovery. The figure below shows a pipeline for integrating data across multi‐omics for evaluation of nucleic acid therapeutics. Inputs from patients based on genomics, transcriptomics, plasma proteomics, and metabolomics are integrated into a virtual tissue model comprising liver, kidney, and immune systems, using information from a single‐cell RNA‐seq atlas and its spatial arrangement. The multi‐omics approach enables simulations to predict target‐knockout effects, off‐target interactions, cytokine secretion, and metabolic disruption. The simulation outputs include predictions of efficacy and toxicity scores, as well as cell‐vulnerability maps.

Multi‐Omics Factor Analysis (MOFA) identifies both shared and omics‐specific variation across datasets such as the transcriptome, proteome, and metabolome. It extracts latent factors that identify biological patterns linked to disease mechanisms and drug responses (Argelaguet et al. 2018). iCluster performs integrative clustering across multiple omics datasets to identify molecular subtypes. It groups samples based on concordant perturbations observed across different molecular layers and discovers biologically meaningful disease subgroups (S. Kim et al. 2017). DIABLO integrates multiple omics datasets while maximizing correlations between them. This approach identifies coordinated molecular signatures across platforms and can also highlight samples showing discordant patterns between omics layers (A. Singh et al. 2019). These computational integration approaches are compiled in Table 4. The general integrated multi‐omics pipeline for mechanistic inference and biomarker discovery for the nucleic acid therapeutic assessment is represented in Figure 4. As can be seen from the scheme, the process begins with the parallel collection of multi‐omics data from the treated biological sample: transcriptomics (bulk RNA‐seq/scRNA‐seq), proteomics (DIA‐MS/DDA‐MS), and metabolomics (LC–MS/GC–MS) with further data preprocessing and quality control. At the level of data integration, unsupervised approaches such as MOFA and DIABLO (Table 4) are applied to detect cross‐omics latent factors that capture biological variance across datasets. Cross‐omics latent factors are then mapped to biological knowledge networks (KEGG, Reactome, WikiPathways) using network‐based integration (Table 4). At the level of predictions, machine learning algorithms are trained on integrated multi‐omics features to predict drug candidates and biomarkers. Figure 4 shows how biomarker hypotheses identified through *in silico* analyses are validated and improved across different stages of drug development within the translational workflow described in Section 7.

**TABLE 4:** Computational integration approaches for multi‐omics analysis in NATs.

Method	Principle	Strength	Application	References
MOFA	Identifys shared variation across omics datasets	Captures hidden biological factors	Drug response prediction	Argelaguet et al. (2018)
DIABLO	Integrates correlated multi‐omics features	Detects coordinated signatures	Biomarker discovery	Singh et al. (2019)
iCluster	Multi‐omics clustering	Identifys disease subtypes	Precision medicine	Kim et al. (2017)
Network analysis	Maps omics changes onto biological pathways	Mechanistic interpretation	Pathway identification	Baião et al. (2025)
Machine learning	Predictive modeling using large datasets	Early toxicity prediction	Drug candidate screening	Baião et al. (2025)

### 6.2 Network‐Based Integration

Network‐based approaches integrate protein–protein interactions, metabolic pathways, and gene regulatory relationships into unified biological networks. These drug‐induced molecular changes can be mapped onto these networks. This strategy helps identify key hub genes and affected pathways (P. Du et al. 2024). Incorporating prior biological knowledge from curated databases such as KEGG, WikiPathways, and Reactome improves the interpretation of multi‐omics results. These knowledge‐based frameworks allow comparison with known therapeutic mechanisms and biological pathways (Ryan et al. 2025).

### 6.3 Causal and Perturbational Modeling

Frameworks for causal inference help determine the direct impact of molecular perturbations and responses. This framework will help identify unexpected pathway activation or hidden mechanisms (Porcu et al. 2021). The perturbation modeling approach is concerned with understanding how the effects of molecular perturbations propagate through signal transduction or metabolism. Predicted responses are compared with multi‐omics data. This can be used to predict responses from multi‐omics data and to discover unexpected pathways or hidden mechanisms (Gavriilidis et al. 2024).

### 6.4 Machine Learning and Predictive Modeling

Machine learning algorithms trained with multi‐omics data from known drugs will help predict and enhance the effectiveness of novel drug development. Some of the features used by these machine learning algorithms include pathway enrichment scores, network centrality, and similarity to molecular signatures (M. Lin et al. 2025). Unsupervised machine learning algorithms will help identify unusual molecular profiles, which suggest the onset of off‐target effects or toxicity. These symptoms will occur earlier, before the conventional clinical markers appear (Bijlani et al. 2022).

## 7 Multi‐Omics Study Design and Translational Pipeline for Drug Discovery

### 7.1 Harmonized Sample Collection and Preprocessing

Biological Replication: A minimum of 3–6 biological replicates per condition provides sufficient statistical power to detect significant omics changes, helps capture rare events, and reduce false negatives in RNA‐seq and similar assays (Schurch et al. 2016; P. P. Singh and Benayoun 2023).

Longitudinal Sampling: Time‐course studies with pre‐dose, early (6–24 h), peak (2–7 days), and chronic (2–4 weeks) time points capture changing pathway responses and help distinguish primary effects from secondary responses (Metwally et al. 2022; Taheriyoun et al. 2026).

Cross‐Tissue Profiling: Combined profiling of the primary target tissue (e.g., liver for hepatotropic therapeutics), secondary nontarget sites, and blood samples allows complete analysis of biodistribution and toxicity (Canzler et al. 2025; Shi et al. 2025).

### 7.2 Bridging Species Differences Using Multi‐Omics and Human Organoid Models

One of the primary shortcomings of rodent‐based preclinical studies for nucleic acid drugs is the divergence between the immune‐sensing apparatus. Specifically, the TLR protein family responsible for sensing PSd ASOs and contaminating double‐stranded RNA in mRNA‐LNP formulations, relative to that found in humans. For instance, the recognition specificity of murine TLR7 and TLR8 differs from that of their human homologs, such that a nucleic acid that is nontoxic to the mouse immune system in hepatic cells may nonetheless trigger IFN‐α/β or inflammatory cytokine production in human immune cells. Such disparities lead to underestimations of immunotoxic signals in routine rodent‐based studies, resulting in unanticipated failures in the clinic based on preclinical data. The use of multi‐omics approaches, such as scRNA‐seq and plasma proteomics, is a promising approach to addressing this issue (Gros and Casanova 2023; Zschaler et al. 2014).

Human liver organoids, liver‐on‐a‐chip platforms, and immune‐competent organoids are increasingly emerging as physiologically relevant human models for evaluating the efficacy and safety of NATs through multi‐omics approaches. Patient‐specific liver organoids derived from human induced pluripotent stem cells (iPSCs) precisely mimic human liver biology, including the TLR expression profile, bile acid biosynthesis, and cytochrome P450 activities, and enable omics profiling of transcripts, proteins, metabolites, lipids, and the spatial distribution of biological components. PRISMA‐compliant systematic review of organoid omics revealed that human liver organoid multi‐omics successfully mimicked pathophysiology of clinically important mechanisms of drug‐induced liver injury, such as mitochondrial dysfunction, endoplasmic reticulum stress, cholestasis due to bile acids abnormalities, and liver damage due to immune response‐all of which are potential toxicity mechanisms of nucleic acid‐based drugs. Importantly, biomarker candidates identified by organoid omics, including bile acid profile, oxidative stress‐response signature, and microRNA profile in extracellular vesicles, corresponded to human hepatotoxicity biomarkers (Ahmad and Muthu Mohamed 2026; Harrison et al. 2021; Q. Liu et al. 2026).

Species bridging using the comparative multi‐omics approach can be achieved through the following procedure. In a comparative study, the same set of chemotypes of ASO/siRNAs is characterized simultaneously in the following settings: (i) mouse and rat primary hepatocytes or rodent tissues; (ii) human primary hepatocytes (PHHs); and (iii) iPSC‐hepatocyte or liver organoids. Then, the signatures of the transcriptome, proteome, and metabolome are compared across these three systems using orthologous gene matching and pathway analyses. Pathways whose activity is consistently activated across all three settings (HMGB1‐mediated sterile inflammation, LDLR pathway modulation by PCSK9 antagonists, and ASOs) may represent conserved, translatable signals. However, immune activation signatures in PHHs and organoids, which are not observed in rodent hepatocytes (e.g., human‐specific TLR8‐mediated activation by single‐stranded RNA motifs), should be viewed as human‐specific liabilities that rodent models missed. Spatial transcriptomics adds an extra dimension to the analysis by revealing zonation of toxicity signals in the periportal and pericentral areas of organoids and liver explants (Gröger et al. 2016; Gros and Casanova 2023; Q. Liu et al. 2026; Wu, Yang, et al. 2025; Zschaler et al. 2014).

It is now possible to perform immune‐competent co‐culture of human PBMCs or liver‐resident immune cells (Kupffer cells, NK cells) with hepatic organoids derived from the same individual, making possible an accurate in vitro model of human autologous immune‐hepatocyte interaction that cannot be achieved in xenograft mice. Single‐cell multi‐omics (CITE‐seq, scATAC‐seq) analysis of these co‐cultures will enable simultaneous resolution of the transcriptional and chromatin landscapes of human hepatocytes, macrophages, and innate immune cells, allowing species‐specific immune response analysis at the cell‐type level. This technique can be directly used to determine whether ASO sequences or the LNP formulation elicit TLR7/8 activation in human monocyte‐derived macrophages/dendritic cells at sub‐hepatotoxic doses, thereby enabling elimination of immunogenic chemotypes before the initiation of the expensive GLP toxicology experiments in animals. The advent of an immune‐competent organoid co‐culture system has enabled co‐culture of human PBMCs or liver‐resident immune cells (Kupffer cells, NK cells) with the corresponding hepatic organoids isolated from the same individual to study autologous human immune‐hepatocyte crosstalk, which is not feasible in xenograft mice. The use of single‐cell multi‐omics approaches (CITE‐seq, scATAC‐seq) on such co‐cultures would help elucidate the gene expression and chromatin changes occurring simultaneously in human hepatocytes, macrophages, and innate immune cells, thereby creating an immune response landscape that is species‐ and cell‐type‐specific. It has potential application in the discovery of ASO sequences or LNPs that activate TLR7/8 in human monocyte‐derived macrophages/dendritic cells without causing hepatotoxicity at sub‐hepatotoxic dose levels (Gröger et al. 2016; Li et al. 2025; Papp et al. 2024).

The application of multi‐omics profiling of human organoids, together with predictive modeling approaches such as causal perturbation modeling and network propagation, enables the prediction of how molecular features arising from organoids will manifest in vivo in human tissues. The use of this human organoid‐based multi‐omics species bridging approach is thus a key step forward toward addressing the long‐standing translational challenge faced by NATs research.

### 7.3 Multi‐Omics Biomarker Signatures for Outcome Prediction

Repeated multi‐omics profiling in humans and well‐characterized animal models shows that combined molecular signatures can decide the therapeutic responses more accurately than single‐omics biomarkers. Combining information from multiple omics layers provides a more detailed view of biological responses to treatment (L. Chen et al. 2025; Jiang et al. 2025). In NATs:

Efficacy Prediction: Early transcriptomic responses, like target knockdown and changes in on‐target pathways, can indicate treatment effectiveness. When these changes are supported by related proteomic and metabolomic changes within 2–7 days, they strongly predict eventual therapeutic efficacy. The lack of target knockdown or activation of compensatory pathways is often linked with clinical treatment failure (Jiang et al. 2025).

Safety Risk Scoring: Multi‐omics toxicity signatures identified in preclinical studies can be used to develop risk scores that predict potential adverse events in clinical settings. For example, early activation of TLR and innate immune pathways in preclinical models indicates inflammatory responses in humans. The hepatic stress signatures may indicate potential ALT elevation, while renal stress markers may predict proteinuria or increased creatinine levels (Bergen et al. 2025; Fogal et al. 2024).

Embedding multi‐omics into the translational pipeline for NATs requires explicit definition of decision points across preclinical and early clinical development. In discovery and lead optimization, omics signatures can be used to rank chemotypes by on‐target efficacy, off‐target burden, and early stress responses in relevant tissues. During preclinical development, longitudinal multi‐omics profiling across species helps determine whether efficacy and toxicity signatures are conserved and whether any species‐specific liabilities may limit human translation. Early‐phase clinical studies offer an opportunity to validate preclinical signatures by serially sampling blood‐based transcriptomic, proteomic, and metabolomic biomarkers alongside standard safety labs. Concordance between predicted and observed signatures supports dose escalation and patient stratification, whereas divergence can trigger redesign of chemistries or delivery systems. This pipeline view positions multi‐omics as a core component of evidence generation for regulatory and clinical decision‐making.

### 7.4 Digital Twins and Tissue Modeling

**FIGURE 5:**
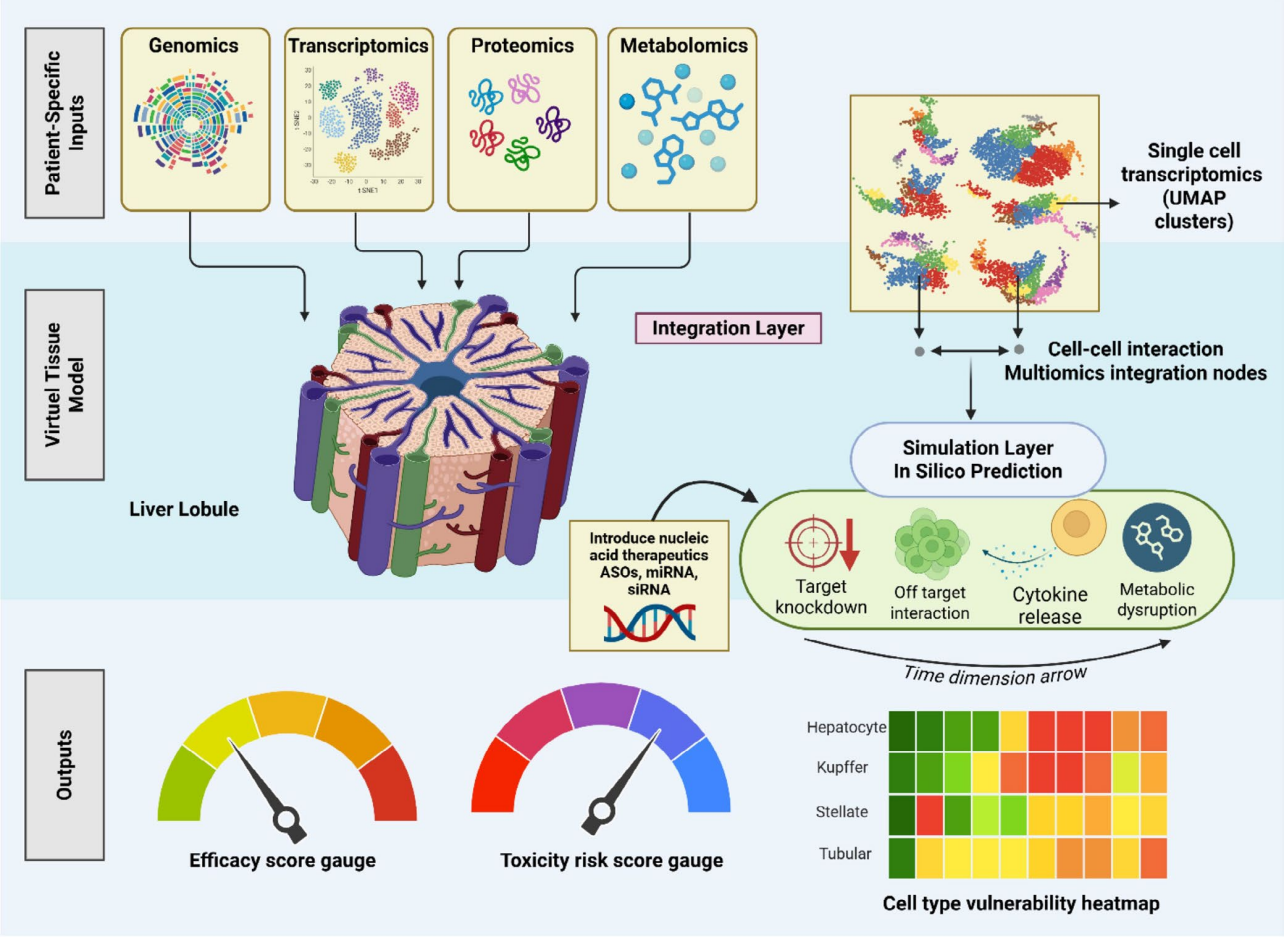
Digital twin framework for predictive modeling of therapeutic response. This figure presents a digital twin approach integrating patient‐specific multi‐omics data with virtual tissue models to predict responses to NATs. Patient‐derived inputs, including genomics, transcriptomics, plasma proteomics, and metabolomics, are incorporated into a virtual tissue model comprising liver, kidney, and immune compartments, informed by single‐cell RNA‐seq atlases and spatial organization. Multi‐omics integration enables simulation of therapeutic perturbation, predicting target knockdown, off‐target interactions, cytokine release, and metabolic disruption over time. Outputs include efficacy and toxicity risk scores, along with cell‐type‐specific vulnerability heatmaps, supporting in silico prediction of therapeutic outcomes and precision medicine applications.

Emerging approaches combine multi‐omics data to create digital twins of tissues. These models integrate different layers of biological information. An overview of this digital twin framework, integrating patient‐specific multi‐omics data with virtual liver, kidney, and immune compartments to predict efficacy and toxicity, is shown in Figure 5. scRNA‐seq creates detailed maps of target tissues, such as the liver or kidney. It also identifies various cell types (e.g., hepatocytes, cholangiocytes, fibroblasts) and different states (quiescent, proliferating, stressed). These maps identify diversity, such as rare subpopulations, that is missed by bulk profiling (Aizarani et al. 2019; Suoangbaji et al. 2024; Rocque et al. 2021). Bulk proteomics/metabolomics measures overall changes at the example tissue level, such as posttreatment pathway flux or protein abundance changes. However, techniques such as single‐cell spatial proteomics (e.g., CyTOF, NanoPOTS) and metabolomics (e.g., SCiMS) can identify cell‐specific responses, such as off‐target immune activation in macrophages or metabolic changes in stressed hepatocytes. This data can be combined with scRNA‐seq maps, enabling researchers to better understand how cells function, how cells interact, and how therapies work across tissues (Allam and Coskun 2024; T. Hu et al. 2023; Taylor et al. 2021). Mechanistic models describe cell interactions, intercellular signaling, and metabolic pathways within tissues. These digital models simulate how genetic changes are introduced by ASO, siRNA, or mRNA therapies and are transmitted through cellular interaction networks and metabolic pathways (Myers et al. 2021). These models help identify vulnerable cell populations before clinical toxicity appears. They help in identifying combinations of therapies that can target compensatory pathways. Therefore, also support the classification of patients based on their likely response and safety profiles (Jafari et al. 2022; Nilsson et al. 2022).

## 8 Future Directions and Emerging Opportunities in Multi‐Omics‐Enabled Nucleic Acid Drug Design

The goal of modern biology is not only to identify which molecules are present in cells but also to determine where they are located within tissues. Spatial transcriptomics and in situ proteomics can solve this problem by correlating cellular information with its location. These techniques enable the observation of cell interactions and the organization of cells in tissues (Chen, You, et al. 2023). Conventional transcriptomic approaches provide comprehensive information on gene expression but do not account for the spatial arrangement of cells during dissociation. The current technology is aimed at preserving the organization of the tissue and studying the patterns of RNA expression with high spatial resolution (around 10–100 μm). Some of the available tools that make it possible include Visium from 10× Genomics (Table 3). Spatial transcriptomics technologies enable the study of gene expression across complex tissues. In cancer research, the method helps determine the spatial organization of both tumor and immune cells. In contrast, in regenerative medicine, it enables understanding of the tissue microenvironment responsible for regeneration, differentiation, and/or fibrosis. While spatial transcriptomics focuses on RNA, in situ proteomics interrogates the functional molecule responsible for most cellular activities that is the protein. With multiplexed imaging, it is possible to detect tens to hundreds of proteins within intact tissue sections using fluorescence labeling or mass spectrometry imaging (Chen, You, et al. 2023; Maciejewski and Czerwinska 2024). Both spatial transcriptomics and in situ proteomics help us understand how our tissues behave by providing more information about the links between molecular profiles and their spatial contexts. They help us better understand tissues and have great potential to improve precision medicine. The recent development of omics technologies enables tracking biological changes not at a single time point but in real time. Real‐time or longitudinal omics monitors changes over time and can provide many interesting facts about disease progression, treatment, and the interaction of our body.

Liquid biopsies are one of the best ways of real‐time molecular analysis that do not require any invasive methods. Instead of using tissue samples for analysis, liquid biopsy analyzes markers in body fluids such as blood, saliva, or urine. The use of circulating cell‐free DNA (cfDNA), extracellular vesicles, RNA, and circulating tumor cells gives us information about current biological processes in our tissues. Tumor‐derived cfDNA fragments provide information about genetic mutations and tissue damage and allow earlier detection of diseases, treatment response, and emergence of drug resistance (Chen, Zhang, et al. 2023; W. Guo et al. 2026).

Continuous monitoring of the human microbiome has recently become a topic of rising interest. Metagenomics and metabolomics methodologies allow scientists to study changes in microbial populations, especially in relation to therapies or diets. These changes can influence drug metabolism, immune responses, and therapeutic outcomes. Differences in gut microbiota could be the reason for increased toxicities or treatment inefficacy in certain patients (Saha et al. 2025; Zhao et al. 2023). In combination with liquid biopsies, microbiome analysis enables continuous monitoring of patients' molecular profiles. Multi‐omics data are becoming increasingly complex and extensive, and AI and ML techniques are necessary to integrate diverse types of information and uncover new patterns and insights. These methods can identify connections between features and differences in biological responses (Zitnik et al. 2019). Graph Neural Networks represent biological systems as networks of connected genes, proteins, and pathways. This technique allows investigation of the impact of changes in a single component on the whole system, rather than examining molecules in isolation. That is why GNNs can be used to predict disease mechanisms, drug effects, and pathway interactions. Foundation models trained on biological and chemical databases can discover patterns in molecular sequences and structures (Deng et al. 2026). Biological and chemical data can be used to train large‐scale foundation models that can learn patterns within molecules. Such models help predict interactions between new molecules and proteins, assess toxicity risks, and support drug discovery (Guo, Guan, et al. 2025). Beyond data analysis, AI systems can also name unexpected correlations within large multi‐omics datasets and generate new research hypotheses. For example, machine learning algorithms may detect links between metabolic changes and gene expression changes in a disease state, suggesting previously unknown regulatory pathways (Yetgin 2025). AI is improving biomedical research by allowing us to predict and understand complex biological systems better than traditional methods.

### 8.1 Regulatory Submission of Multi‐Omics Data and Analytical Pipeline Standardization

With the growing significance of multi‐omics in the characterization of NATs at preclinical and early clinical stages, an important issue is the data submission, review, and regulatory acceptance of these complex datasets. At present, the FDA expects study data submissions in standardized electronic format, using SEND and CDISC forms for nonclinical and clinical studies, respectively, for NDAs, BLAs, and INDs. Furthermore, there are FDA Study Data Technical Conformance Guides and other guidance materials that provide metadata and review guidelines for datasets. However, there are no established regulatory requirements for the format and analysis of multi‐omics datasets, including transcriptomics, proteomics, metabolomics, and single‐cell omics, for IND/NDA submissions (CDISC; FDA). Several issues arise because of the mentioned gap. To begin with, there is no established minimum standard for an acceptable multi‐omics dataset that could be evaluated through the regulatory process, e.g., depth of coverage, number of biological replicates, and the statistical significance threshold required to make a claim of safety or pharmacodynamics. Second, the computational analysis pipelines used for data preprocessing, normalization, batch correction, and pathway enrichment vary widely across labs and are therefore difficult to evaluate for reproducibility. Third, there are practical difficulties connected with the huge file sizes of raw omics data (especially scRNA‐seq and spatial transcriptomics). As far as New Approach Methodologies (NAMs), including organoid‐based omics, are concerned, the FDA Modernization Act 2.0 promotes the adoption of alternatives to animal testing; however, standards such as SEND and CDISC can provide a framework, while reporting standards for organoid‐based multi‐omics are being developed (CDISC 2026; Vo and Le 2026).

Some useful pathways are starting to take shape. The FAIR data principles, now promoted by many funding agencies and cited in EU‐ToxRisk and similar programs, provide an approach to multi‐omics data governance that is consistent with requirements for traceability, reproducibility, and achievability. Multi‐omics data submissions according to FAIR guidelines will need structured metadata, ontological annotations according to standard ontologies (such as Gene Ontology, ChEBI, and MeSH controlled vocabularies), pipeline descriptions along with version‐controlled software and containerized pipelines (such as Nextflow, Snakemake), and deposits in publicly available archives such as NCBI GEO, ProteomeXchange, or MetaboLights. Furthermore, in the FDA and EMA joint principles on AI evidence generation (published January 2026), it is specified that any AI or machine learning application to multi‐omics data for regulatory purposes must be done in a well‐defined context of use, with proper data governance, and with audit trails. This is also applied to ML‐based multi‐omics integration applications such as MOFA and DIABLO, as well as to pathway enrichment methods for nucleic acid therapeutic evaluation (EMA‐FDA; 2026; Mugahid et al. 2025; Vo and Le 2026).

The recommendations below could serve as a basis for sponsors aiming to use multi‐omics technology in their drug development process. First, an early interaction with the FDA or EMA at a pre‐IND meeting or scientific advice could help clarify regulatory expectations regarding the types of omics data expected. Second, omics data should be collected in line with the FAIR principles, and raw data should be submitted to suitable public repositories when possible. Third, analysis needs to be done using a validated computational pipeline with a fixed software version. Fourth, omics assays need to include relevant internal positive controls, like well‐known hepatotoxic compounds. Fifth, regulatory submissions should highlight pathway‐level analysis and summary statistics rather than just raw count matrices. In conjunction with emerging guidelines from regulatory agencies on the submission of omics‐heavy applications (likely to follow the FDA's voluntary pharmacogenomic submission guidelines), the NATs community needs to take a proactive role in defining scientific and realistic standards (CDISC 2026; EMEA‐FDA 2026; FDA 2026; Mugahid et al. 2025).

## 9 Conclusion

Multi‐omics approaches such as proteomics, metabolomics, and single‐cell transcriptomics play a key role in evaluating NATs. Through simultaneous examination of the transcriptome, proteome, and metabolome at a single cell resolution, investigators will be able to differentiate between real drug effects and off‐target effects or toxicities. This approach would optimize treatment based on molecular response classification. Proteomics directly indicates target engagement, pathway modulation, and early signs of stress. Metabolomics supplements the information obtained through proteomics by showing metabolic alterations due to pathway modulation and early signs of toxicity. Single‐cell transcriptomics can help address the issue of cell‐type‐specific responses and detect rare populations of cells responsible for adverse effects and even immune activation that could not be seen otherwise. Using these types of data together with advanced computational tools, it becomes possible not just to observe but to gain a better understanding of the mechanism of action of therapeutics. Such an understanding plays an important role in developing precision medicine. To accelerate clinical implementation of NATs, multi‐omics validation needs to become a part of the preclinical development process. This approach will help detect issues related to off‐target effects, immune response, and organ toxicity that can limit treatment efficiency and patient eligibility. Full utilization of NATs will require not only continued funding for the infrastructure for multi‐omics and standardization of experimental and computational approaches but also training of researchers in systems biology. Cooperation between academia, industries, and regulators will play a key role in this regard.

## Author Contributions

**Gurjit Kaur Bhatti:** conceptualization, investigation, writing – original draft, writing – review and editing, visualization, validation, methodology, formal analysis, project administration, supervision, resources, data curation. **Anushka Verma:** investigation, writing – original draft, writing – review and editing, visualization, validation, methodology, formal analysis, data curation. * Umesh Chand Singh Yadav5: conceptualization, investigation, writing – original draft, visualization, writing – review and editing, validation, formal analysis, supervision, data curation, project administration. **Jasvinder Singh Bhatti:** conceptualization, investigation, funding acquisition, writing – original draft, writing – review and editing, visualization, validation, methodology, software, formal analysis, project administration, resources, supervision, data curation. **Komal Devi:** investigation, writing – original draft, writing – review and editing, visualization, validation, methodology, formal analysis, data curation. **Inderpal Singh Sidhu:** writing – review and editing, visualization, formal analysis, supervision, resources, project administration, investigation, conceptualization, writing – original draft, validation. **Naina Khullar:** investigation, writing – original draft, writing – review and editing, visualization, validation, formal analysis, methodology.

## Funding

We acknowledge the support provided by the Anusandhan National Research Foundation (ANRF) to Central University of Punjab under the Partnerships for Accelerated Innovation and Research (PAIR) programme (Sanction order No. ANRF/PAIR/2025/000029/PAIR dated 20 September 2025).

## Ethics Statement

The authors have nothing to report.

## Conflicts of Interest

The authors declare no conflicts of interest.

## Data Availability

Data sharing not applicable to this article as no datasets were generated or analyzed during the current study.
